# Ocean change within shoreline communities: from biomechanics to behaviour and beyond

**DOI:** 10.1093/conphys/coz077

**Published:** 2019-11-18

**Authors:** Brian Gaylord, Kristina M Barclay, Brittany M Jellison, Laura J Jurgens, Aaron T Ninokawa, Emily B Rivest, Lindsey R Leighton

**Affiliations:** 1 Bodega Marine Laboratory, University of California at Davis, 2099 Westshore Road, Bodega Bay, CA 94923, USA; 2 Department of Evolution and Ecology, University of California at Davis, One Shields Avenue, Davis, CA 95616, USA; 3 Earth and Atmospheric Sciences Department, 1-26 Earth Sciences Building, University of Alberta, Edmonton, AB T6G 2E3, Canada; 4 Biology Department, Bowdoin College, 255 Main Street, Brunswick, ME 04011, USA; 5 Marine Biology Department, Texas A&M University at Galveston, 200 Seawolf Parkway, Galveston, TX 77553, USA; 6 Department of Biological Sciences, Virginia Institute of Marine Science, William & Mary, 1370 Greate Road, Gloucester Point, VA 23062, USA

**Keywords:** Behavioural responses, functional ecology, global environmental change, global warming, ocean acidification, species interactions

## Abstract

Humans are changing the physical properties of Earth. In marine systems, elevated carbon dioxide concentrations are driving notable shifts in temperature and seawater chemistry. Here, we consider consequences of such perturbations for organism biomechanics and linkages amongst species within communities. In particular, we examine case examples of altered morphologies and material properties, disrupted consumer–prey behaviours, and the potential for modulated positive (i.e. facilitative) interactions amongst taxa, as incurred through increasing ocean acidity and rising temperatures. We focus on intertidal rocky shores of temperate seas as model systems, acknowledging the longstanding role of these communities in deciphering ecological principles. Our survey illustrates the broad capacity for biomechanical and behavioural shifts in organisms to influence the ecology of a transforming world.

## Teaser

**Lay Summary**: Brief overview of biomechanical and behavioural consequences of ocean acidification and warming for marine organisms and communities inhabiting rocky temperate shores.

## Introduction

Humans are now the greatest agent of ecological change globally. Effects span those due to habitat loss, increased exchange of taxa across space, pollution, altered nutrient cycling, overfishing, reductions in top predators, as well as fundamental shifts in the properties of Earth’s atmosphere and oceans associated with emissions of carbon dioxide (Vitousek et al., 1997; Jackson et al., 2001; Estes et al., 2011). In marine systems, the latter perturbations drive especially important modifications to temperature and seawater chemistry (Feely et al., 2004; Harley et al., 2006; Doney et al., 2009). These physical changes affect biomechanical and behavioural processes relevant to organisms, including ones that alter the ecology of sea creatures and the communities in which they live.

Considerable work documents how marine life is affected by ocean warming and ocean acidification (OA; i.e. human-induced decreases in seawater pH and carbonate ion concentrations accompanied by elevated aqueous CO_2_; Caldeira and Wickett, 2003). Much of this research focuses on responses of individual taxa to these changes, centering on demographically relevant parameters like growth, survival and reproduction. Biomechanical consequences have received less attention. Similarly, implications for interactions amongst species remain underexplored, especially in OA research where studies primarily address effects on single species in isolation (Kroeker et al., 2010, 2013b). Although growing numbers of experiments examine the capacity of OA to alter behaviour (a core component of interactions amongst animals; see, e.g. Dixson et al., 2010; Watson et al., 2013; Jellison et al., 2016; Jellison and Gaylord, 2019), substantial knowledge gaps persist. Studies addressing effects of rising temperature incorporate a deeper functional and ecological perspective, but also highlight mostly shifts in demography and distribution (Poloczanska et al., 2013). Examinations of how environmental change may alter biomechanical and behavioural attributes of organisms, and accompanying relationships amongst taxa remain limited.

Effects of global change, however, are most apparent when the form, function and performance of organisms are evaluated in the context of the networks of species connections present within communities. Even when implications of altered temperature or chemistry apply most obviously to individual species, linkages amongst taxa pertain (Gaylord et al., 2015). Mechanism at the organismal scale influences species interactions at the ecological scale, with broader implications for natural systems. Indeed, marked ecological transitions associated with climate change are often mediated through shifts in organism-level traits and species interactions. Such ecological shifts do not necessarily arise just because a particular species exhibits susceptibility, but because the interplay between that species and others becomes disrupted, sparking an amplified response. Non-linearities intrinsic to such perturbations create thresholds, tipping points and even ecological phase shifts, where a system flips to another state and manner of functioning (Scheffer et al., 2001). Examples include warming-induced range expansions in sea urchins that induce widespread kelp deforestation (Ling, 2008; Ling et al., 2015), coral bleaching driven by high ocean temperature that causes ecological phase shifts in reef fish assemblages (Bellwood et al., 2006), and trophic cascades in rocky bottom communities deriving from climate-associated disease outbreaks in echinoderms (Schultz et al., 2016; Harvell et al., 2019).

**Figure 1:**
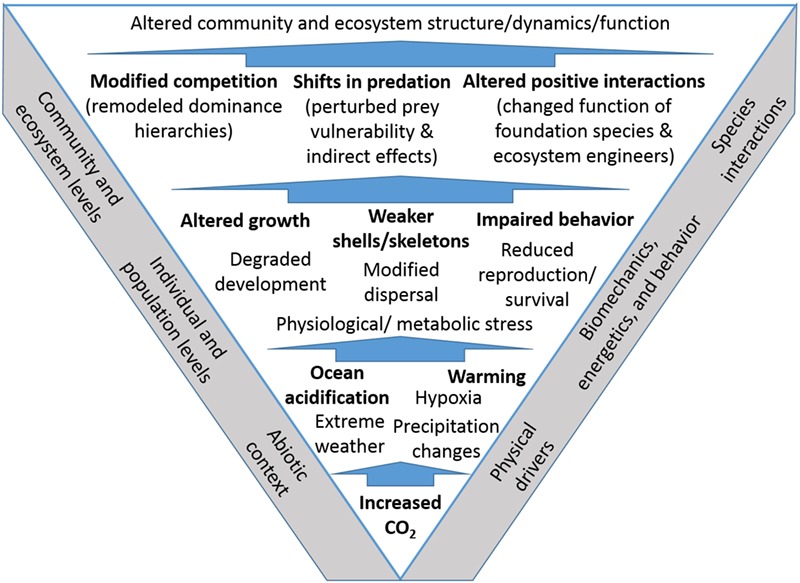
Conceptual diagram highlighting how effects of elevated emissions of carbon dioxide (CO_2_) can cascade upwards from lower levels of biological organization, through interactions amongst species, to ultimately influence community and ecosystem-scale properties. Although common consequences of elevated CO_2_ are depicted, they are not exhaustive; subjects shown in bold are explored in this review.

Here, we address additional examples where biomechanical and behavioural consequences of environmental change link responses of organisms at the individual level to broader ecological concerns. We focus on temperate rocky intertidal zones, where physical–biological processes have been studied for decades, and where important ecological concepts have been elaborated. For the purposes of our overview, we discuss the current state of knowledge regarding climate stressors in such habitats, with more in-depth consideration of examples from our own studies. We focus especially on benthic stages of organisms, while also incorporating insights from larval studies given the importance of these dispersing stages for population structure and dynamics (e.g. Gaylord and Gaines, 2000; Gaylord et al., 2006; O’Connor et al., 2007; Byrne and Przeslawski, 2013; Nickoks et al., 2015). Our framework for discussion accounts explicitly for the capacity of biomechanical and behavioural traits of organisms to translate impacts through species interactions and drive broader changes in community and ecosystem properties (Fig. 1).

## Environmental change and rocky shores

Intertidal habitats on open coasts experience dramatic swings in temperature and desiccation state as the tides rise and fall, as well as large forces imposed by rapid water velocities (see Denny and Gaylord, 2010; or Denny, 2016 for a biomechanical perspective). These challenges are exacerbated as climatic warming and shifts in wind patterns produce stronger thermal forcing and bigger ocean waves in some areas (Young et al., 2011; Reguero et al., 2019). OA operates as an additional, emerging threat (Orr et al., 2005; Kroeker et al., 2013a; Gaylord et al., 2015; Nagelkerken and Connell, 2015).

The physical stresses of rocky intertidal habitats also provide a backdrop for processes of predation, competition and facilitation that further define the structure and dynamics of these communities, and which have been well studied (see also Gaylord et al., 2015). Indeed, the importance of predation and herbivory (Paine, 1969; Lubchenco, 1978; Power et al., 1996), roles of competition and disturbance (Connell, 1961; Dayton, 1971; Sousa, 1979), the capacity for recruitment and environmental stress to modulate effects of predation and competition (Menge and Sutherland, 1987), all have been researched intensely in rocky intertidal environments. Understanding of facilitation (Stachowicz, 2001; Bruno et al., 2003) and habitat provisioning by foundation species (Dayton, 1972), or habitat modification by ecosystem engineers (Jones et al., 1994), has likewise benefitted from research conducted in shoreline communities (Lafferty and Suchanek, 2016; Jurgens and Gaylord, 2018).

The close connection between physical attributes of intertidal environments, the functional traits of organisms (including biomechanical and behavioural ones), and key ecological processes is also complicated by the geographic variation that exists in thermal and chemical stressors active within these areas. For instance, latitudinal gradients in solar input combine with variation in the timing of low tides to create mosaics of temperature extremes in intertidal habitats along the US west coast (Helmuth et al., 2002, 2006). Analogous mosaics in OA-related conditions arise due to regional variation in prevailing winds and oceanographic responses to those winds (Feely et al., 2008, 2016; Chan et al., 2017). Such variation, and accompanying differences in species composition across space (Abbott and Hollenberg, 1976; Morris et al., 1980; Carlton, 2007; see also Sanford et al., 2019), creates strong impetus to understand how organisms interact within the community assemblages that characterize a given location within the broader coastal landscape.

## Ocean perturbations and biomechanics of predation

Amongst the most obvious ecological consequences of altered temperature and seawater chemistry are those tied to animal feeding and consumption. Energetic, biomechanical and behavioural considerations apply, of which we begin with the first two. Temperature affects physiology, including metabolic rate, energy demands, and often the impetus for, and intensity with which, marine animals forage (Schmidt-Nielsen, 1997; Hoffmann and Todgham, 2010; Somero, 2010). From the perspective of prey, seawater chemistry—in particular properties tied to the carbonate system and saturation state of calcium carbonate—influences the ability of calcifying taxa to precipitate shells, spines and skeletons (Ries et al., 2009). Such structural elements are essential features of predator deterrence (Vermeij, 1982).

There are clear examples where ocean change may impinge on processes underlying predation, including those affecting key community members. Consider California mussels (*Mytilus californianus*), which reach high abundances and operate as a major space holder in mid-intertidal regions on rocky shores of the eastern Pacific Ocean. They are often viewed as a competitive dominant amongst larger macro-fauna (Paine, 1966). At the same time, the complex, 3D matrix of the beds formed by this mussel provides habitat and shelter for hundreds of other species (Suchanek, 1992; Lafferty and Suchanek, 2016). Thus, this taxon serves as an ecosystem engineer that facilitates the success of many other organisms, making factors that influence its susceptibility to predation highly relevant to the community.

**Figure 2:**
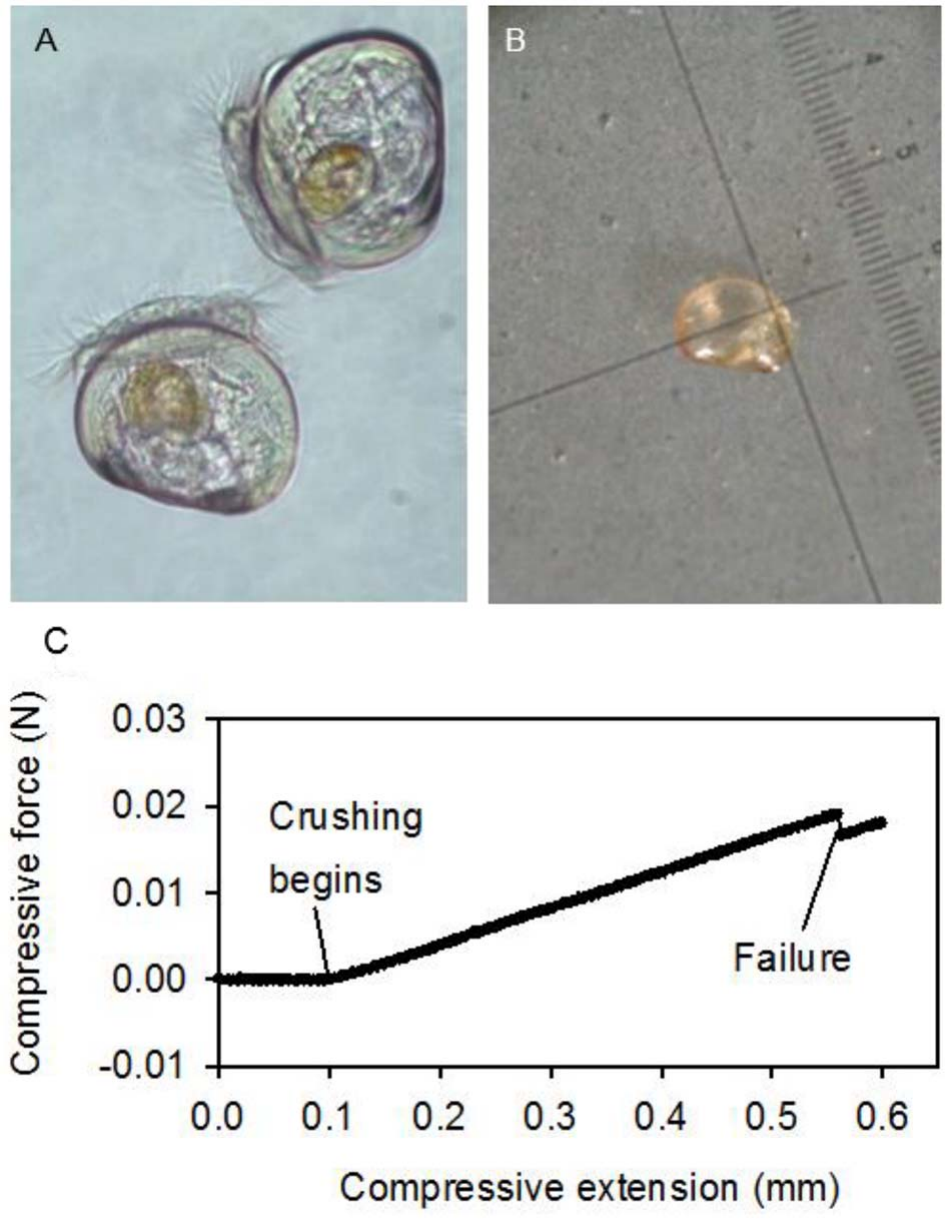
Biomechanical properties of the shells of larval mussels (*Mytilus californianus*) can be tested shortly before settlement into the benthos, as a function of their exposure to climate change drivers. (A) Veliger stage mussel larvae. The long axis of each of these individuals is ~120 μm. (B) Individual larvae can be mounted on microscope slides and the compressive force required to break their shells quantified, using a micro-force applicator affixed to a materials testing instrument. Smallest scale division equals 12.5 μm. (C) Example force trace during a representative test. Data and images redrawn after Gaylord et al. (2011).

**Figure 3:**
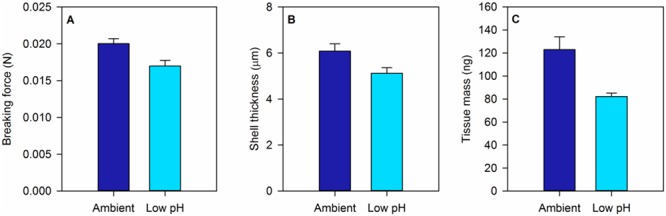
OA can cause late-stage mussel larvae (*Mytilus californianus*) to produce weaker and thinner shells, along with bodies of reduced tissue mass. Ambient seawater conditions were characterized by a pH of 7.9 (quantified on the total scale), and low-pH conditions by a pH of 7.6. Error bars indicate SEM. Further experimental details and full dataset can be found in Gaylord et al. (2011).

Our research and that of others implicates OA and elevated water temperatures as agents by which the biomechanical vulnerability of California mussels to consumption may change. In laboratory experiments, larval mussels exposed to elevated-CO_2_ (or lower-pH) seawater precipitate smaller shells, as well as shells that are thinner and weaker. These effects are revealed through standard imaging techniques such as scanning electron microscopy and classic biomechanical tests of breaking strength (Figs 2 and 3; Gaylord et al., 2011). Moreover, larval mussels cultured under OA conditions also produce less internal tissue for their body size (Fig. 3). Analogous experiments by Frieder et al. (2014) recapitulate a number of these trends, as do field studies conducted by our group concerning newly settled larval recruits. Together these experiments indicate the potential for altered interactions of young mussels with predators in the face of ocean change. Because larval mussels retain their shells when they complete their pelagic larval phase and settle into the benthos, degraded shell integrity or reduced body mass may impose a suite of costs on small juveniles. For example, new settlers must survive attacks by crushing and drilling predators that inhabit the beds formed by adult mussels (Gosselin and Qian, 1997). Shells weakened by OA may be less effective at resisting the chelae of the many juvenile crabs, for instance, that live within the matrix of mussel beds. Similarly, thinner shells may require less time and effort for carnivorous, drilling gastropods to penetrate (e.g. Kroeker et al., 2014b), an effect that may be exacerbated in warmer conditions (Miller, 2013). Decreased tissue mass could encourage higher attack rates by consumers, by decreasing the energetic reward associated with a given mussel individual and thereby driving predators to consume more individuals per time (Sanford et al., 2014).

Negative effects of OA on shell properties may also apply to older and larger stages of mussels. Reciprocal transplants with mid-sized juvenile *M. californianus* across multiple sites along the west coast of North America show elevated susceptibility to predation by drilling whelks in regions of greater OA stress, if high levels of food are unavailable, or in especially warm locations (Kroeker et al., 2016). Moreover, shell thickness of large, adult California mussels has decreased over time, such that shells from the present day are significantly thinner than ones collected during the 1970s and from Native American middens dated to 1000–2400 years ago (Pfister et al., 2016). Additional data indicate accompanying changes in mussel shell mineralogy over the past 15 years (McCoy et al., 2018). It would be valuable to know whether frequencies of drill holes from predatory gastropods have risen over time in accordance with such thinning and changes in mineralogical properties.

## Interspecific variation in biomechanical responses

While mussels appear to experience increased susceptibility to predation under environmental change, not all shoreline taxa will respond the same way. Paired work on two common gastropods found in intertidal habitats along the US west coast highlights disparate responses amongst taxa. In a long-term exposure of the herbivorous gastropod, *Tegula funebralis* (the black turban snail), and a predatory counterpart, *Nucella ostrina* (the striped dogwhelk), to OA, Barclay et al. (2019) demonstrate both notably different changes between these two taxa in how shell properties shifted under altered seawater chemistry, as well as cryptic changes that were not obvious by eye.

**Figure 4:**
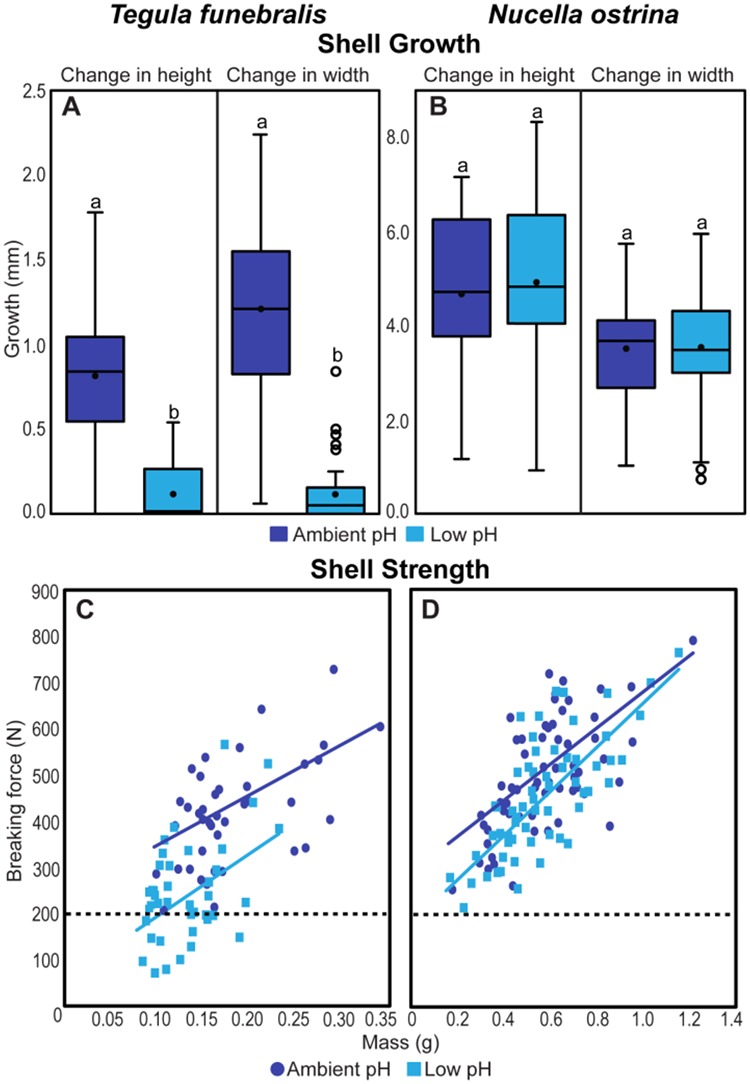
Shell growth and strength of the black turban snail, *T. funebralis*, and the striped dogwhelk, *N. ostrina* after 185 days of exposure to decreased seawater pH (pH ~ 7.4, half a unit lower on the total scale than ambient seawater). Note that the axes differ between left and right panels given the different growth and strength patterns of *Tegula* (left) and *Nucella* (right). (A and B) Boxplots of new shell growth (both height and width) measured over the 185-day experiment (*n* = 40 per treatment). Ambient treatments are in dark blue, manipulated (decreased) pH treatments are in light blue, with boxes indicating upper and lower quartiles, central lines indicating medians, means as black circles, and whiskers representing min/max data. (C and D) Scatterplots of shell strength (*n* = 20 and 30 per treatment for *Tegula* and *Nucella*, respectively). Mass (g) of dried shells was used as a proxy for size. Maximum force (*N*) values indicate forces exerted at the point of total shell failure. Dark blue (ambient pH) and light blue (low pH) trend lines indicate best-fit regressions. The black-dotted line at 200 N indicates conservative crushing forces exerted by adult crabs of *C. productus* (Taylor, 2000). Modified from Barclay et al. (2019).

When *Tegula* and *Nucella* were exposed to reduced-pH seawater for 6 months, the former experienced substantial reductions in shell growth (~88%) and strength (~50%), while the latter experienced no effects on growth and only moderate reductions in strength (~10%) (Fig. 4). In this case, shell strength was measured in response to a compressive load applied perpendicular to the axis of coiling in the manner of a crab pinch. The use of intact shells accounted for failure traits that were dependent on both material properties and shell geometry. As has been shown in analogous systems involving biting consumers where gape dimensions are important (e.g. Dumont and Herrel, 2003), the observed patterns in breaking force, and their relationship to shell growth and size, have ecological relevance. Shells of smaller width and height are susceptible to a broader range of size classes of crushing predators, and declines in shell strength decrease the force required of a predator. Therefore, although both gastropod species will likely become increasingly vulnerable to predation under future ocean conditions, *Tegula* may become substantially more so. At the same time, crustaceans that often dominate guilds of shell-crushing predators, including species such as the common rock crabs, *Cancer productus* and *Romaleon antennarium*, appear less susceptible to OA (Ries et al., 2009; Whiteley, 2011). Such differential responses to shifts in seawater chemistry could skew biotic interactions involving various combinations of predators and prey (Kroeker et al., 2014b).

In addition to shifts in shell size and/or shape, changes in mineralogy and/or microstructure could contribute to shell weakening. In the case of *Tegula* and *Nucella*, these two species precipitate shells that differ in composition. The former has a nacreous shell made of columnar/stacked aragonite crystals, whereas the latter has an inner cross-lamellar aragonitic shell with an outer layer of homogeneous calcite (Geller, 1982; Avery and Etter, 2006). Calcite is chemically more stable than aragonite and less prone to dissolution (Watabe, 1988). The potential for divergent mineralogies to lead to distinct responses to OA is well recognized (e.g. Welladsen et al., 2010; Ries, 2011; Amaral et al., 2012; Coleman et al., 2014; MacKenzie et al., 2014; Wright et al., 2014, 2018; Moulin et al., 2015; Pickett and Andersson, 2015; Dery et al., 2017). Still, because mineralogical responses to shifts in temperature or OA may not be visually obvious, and yet have the capacity to affect shell integrity (and therefore resistance to predation), explicit examinations of shell and skeletal structural properties remain important. Indeed, had Barclay et al. (2019) not included a mechanical test of shell strength, one might have assumed that *Nucella*’s anti-predator defenses were unaffected by OA, given that its macroscopic patterns of growth did not change in low-pH seawater.

The case study of *Tegula* and *Nucella* also underscores additional ways in which environmental change might shift the character of predator–prey interactions. Although further experiments are required, the disproportionate decline in growth and shell strength in *Tegula* under OA implies a potential for crabs to alter how they rank this species as a food source relative to *Nucella*. Both gastropods are frequently prey for shell-crushing crabs, but under normal conditions, *Tegula* is more resistant to predation than similarly sized *Nucella*. In particular, crabs attacking *Tegula* are more likely to fail in the attack, and the handling times for *Tegula* are significantly greater. Nevertheless, crabs regularly target *Tegula*, suggesting that black turban snails are preferred for other reasons (e.g. they may contain more tissue mass for a given size). If such is the case, then under future ocean conditions when breaking strengths of *Tegula* are likely to decline to levels readily managed by crabs, it is conceivable that crabs will pursue *Tegula* with substantially greater vigor than *Nucella*. Because *Tegula* and *Nucella* occupy different trophic levels—with the former herbivorous and the latter carnivorous—changes in relative predation pressure could modify patterns of energy flow through the food web. This potential for environmental change to reconfigure feeding links in marine communities warrants further research.

## Ocean chemistry, behaviour and altered community structure

One of the more far-reaching implications of OA is that chemical changes to seawater can induce marked shifts in animal behaviour (Munday et al., 2009; Dixson et al., 2010; Nilsson et al., 2012; Watson et al., 2013; Jellison et al., 2016; Jellison and Gaylord, 2019; Rivest et al., 2019). Such shifts in behaviour complement those tied to altered biomechanical properties by influencing foraging activities of organisms and their responses to predators. One example of where OA-induced behavioural impairments may have critical implications is in tidepool systems. *Pisaster ochraceus*, the ochre sea star, operates as a major predator along rocky shores of the eastern Pacific Ocean, where foundational work by Paine (1969) highlighted its keystone role in influencing community structure through its consumption of the dominant mussel space-holder, *M. californianus*. However, *Pisaster* and other predatory sea stars such as the six-armed star, *Leptasterias hexactis*, have also been shown to influence community organization not only by eating mussels, but also by inducing widespread flight responses and altered foraging patterns in less preferred prey such as herbivorous gastropods. In this regard, factors that affect how prey responds behaviourally to predatory sea stars could perturb community structure and dynamics.

Laboratory trials indicate the capacity for OA to influence refuge-seeking behaviours and foraging activities of the black turban snail, *T. funebralis*, in response to *Pisaster* and *Leptasterias* sea stars (Gooding et al., 2009; Jellison et al., 2016; Jellison and Gaylord, 2019). *Tegula* is an abundant and conspicuous herbivore along many rocky shores of the US west coast, and operates as a non-trivial contributor to energy transfer through these systems. Therefore, it is of note that *Tegula* snails held in low-pH seawater typical of intertidal rock pools display attenuated predator-avoidance behaviours. Moreover, such behavioural impairments may become more common as OA exacerbates the low-pH conditions that currently derive from the accumulation of respiratory CO_2_ in the pools when they are isolated during night-time low tides (Jellison et al., 2016; Kwiatkowski et al., 2016; Silbiger and Sorte, 2018; Jellison and Gaylord, 2019; also see Bracken et al., 2018).

*Tegula*’s role as a common grazer means additionally that effects of predatory sea stars can extend beyond *Tegula* to influence trophic levels below; in this case, the macroalgae grazed by snails. Such cascading effects can follow two paths. One arises when the sea stars eat *Tegula* individuals, which decreases the number of snails and how much macroalgae gets consumed. Another path derives from the propensity of *Tegula* to behaviourally avoid sea stars, which can reduce foraging by the snails when they detect sea stars (i.e. *Tegula* becomes too fearful to feed). Both these pathways could be altered by low pH. For example, OA-induced impairment of anti-predator behaviour could make *Tegula* snails more vulnerable to predation, increasing their mortality rates and decreasing how many remain to feed on macroalgae. Alternatively, OA could cause atypical boldness in snails, attenuating their flight responses and raising their risk of being eaten, but also fostering elevated foraging and per-capita consumption of macroalgae.

**Figure 5:**
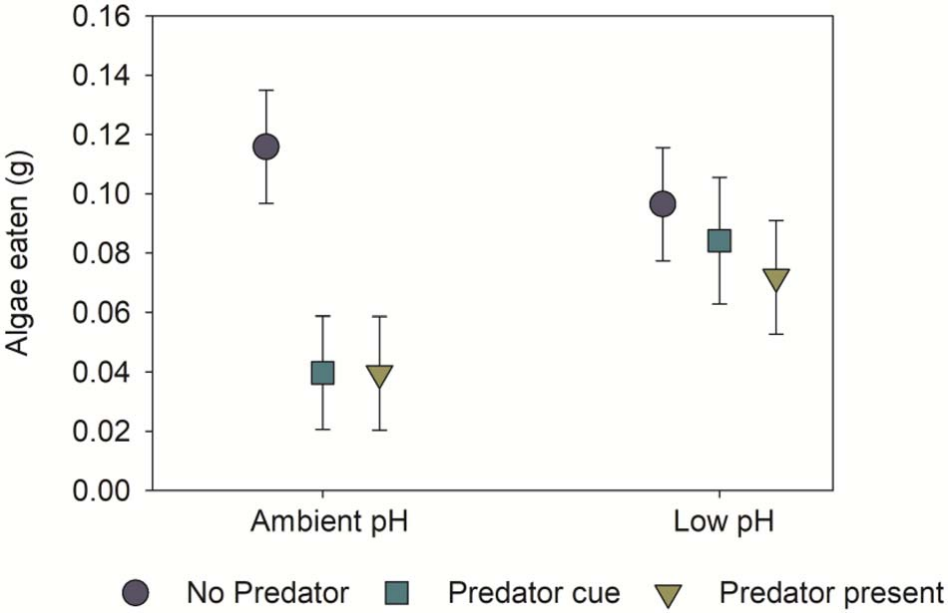
Exposure to low-pH seawater can attenuate the antipredator behaviour of herbivorous snails (*T. funebralis*) that inhabit tidepools, altering their grazing patterns and thus the way in which cascading, top–down effects of sea-star predators operate in the system. Under ambient conditions (pH ~ 7.9 on the total scale) in mesocosm experiments, snails exit the water when they sense the presence of sea-star predators (*L. hexactis*; yellow triangle) or dissolved cue from them (blue–green square); this flight response provides a spatial refuge from predation and reduces the grazing pressure of snails on macroalgae. However, under low-pH conditions (pH ~ 6.9, expected in isolated tidepool waters during nighttime low tides under OA), snails spend more time in the water in the presence of sea stars, increasing their access to and consumption of macroalgae relative to when they are in ambient seawater. Error bars represent 95% confidence intervals. Figure modified from Jellison and Gaylord (2019).

Mesocosm experiments used to explore these dual pathways demonstrate that decreased seawater pH can lead to an overall reduction in the cascading influence of sea stars on macroalgal consumption by *Tegula* snails (Fig. 5; Jellison and Gaylord, 2019). Under contemporary seawater conditions, as snails represent a less preferred prey item for sea stars (Gravem and Morgan, 2019), they are rarely eaten, yet still demonstrate a strong avoidance behaviour and a reduced rate of grazing on macroalgae when the scent of sea stars is present. However, in low-pH seawater, the antipredator behaviour of snails becomes muted, causing them to partially ignore sea-star scent. This outcome results in increased snail feeding relative to that which occurs in ambient seawater containing the aroma of sea stars. Moreover, the increased feeding rates under low-pH conditions are sufficiently elevated that, even though more snails are killed by sea stars, the snails that remain eat more macroalgae overall. By this mechanism, low pH can weaken the positive indirect influence of sea stars on macroalgae.

In addition to OA-induced behavioural alterations that impinge on predator–prey interactions, competitive interactions can shift within communities subjected to perturbed seawater chemistry. McCoy and Pfister (2014) demonstrate shifts in the dominance hierarchy of calcified coralline algae that compete through overgrowth interactions on rocky intertidal shores. In particular, these researchers revealed a reorganization between the 1980s and the present day of the rankings that dictate which species is superior to another in this guild. Although additional work is required to understand the underlying drivers of such changes, these modified competitive interactions could be mediated at least in part through properties or processes associated with biomineralization. Analogous but more dramatic shifts in competitive hierarchies occur in response to OA in tropical reef systems. In these latter cases, weedy, mat-forming algae gain prominence at the expense of other taxa such as corals (Fabricius et al., 2011; Harley et al., 2012; Connell et al., 2013).

## Biomechanics of heat, momentum and mass exchange under global change

As alluded to above, California mussels appear vulnerable to climatic changes. Consistent with this possibility, the abundance and size of the congeners *M. californianus* and *M. trossulus* have declined in northern Washington State, with modelling suggesting a causal link to OA (Wootton et al., 2008). Percent cover of *M. californianus* in Southern California has fallen relative to historical levels (Smith et al., 2006), and the abundance of another species, *M. edulis*, on the US east coast has dropped by >60% since the 1970s (Sorte et al., 2017). The latter two studies implicate temperature increases as a possible driver. Long-term research along the Oregon coast, spanning >14 years, has additionally revealed correlations between large-scale oceanographic indices (in particular, the North Pacific Gyre Oscillation, or NPGO) and mussel recruitment (Menge et al., 2009). These correlations suggest a relationship between climate-driven shifts in ocean dynamics and larval survival, potentially due to NPGO-related changes in phytoplankton food availability. Connections between mussel performance, food and global change factors (including temperature and seawater pH; Juranek et al., 2009; Alin et al., 2012; Davis et al., 2018) are further reiterated by correlations between geographic variation in the strength of coastal upwelling and growth of juvenile mussels. Throughout California and other portions of the US west coast, decreased pH and low food are associated with reduced mussel growth, as are peak aerial temperatures during low-tide emergence (Kroeker et al., 2016). Complementary results from laboratory trials using *M. galloprovincialis* likewise demonstrate declines in growth under OA, with warmer water temperatures offsetting this trend (Kroeker et al., 2014a). O’Donnell et al. (2013) and Zhao et al. (2017) document negative effects of OA on the attachment strength of the byssal threads that allow mussels to adhere to the rock; such responses could further contribute to population decreases in these species.

Given the ecological status of mussels as major space occupiers and providers of habitat for other species and juvenile conspecifics, their impaired performance under OA and warming has strong implications for a variety of interactions in intertidal environments. In what follows we emphasize interactions governed by physical transport and exchange processes that often operate as foci for biomechanical study, and which underpin the capacity for intra- and inter-specific facilitation by mussels. At low tide, dense beds of adult mussels ameliorate stresses for juvenile size classes and other organisms that live within the mussel assemblage. In particular, thermal and desiccation stresses, as controlled by heat transfer and evaporative mass exchange, can be modulated appreciably within *M. californianus* beds, such that temperature and desiccation extremes are substantially attenuated (Jurgens and Gaylord, 2016, 2018). Indeed, thermal extremes are so effectively buffered in the midst of mussel aggregations that typical latitudinal patterns in high-temperature exposure are functionally eliminated (Jurgens and Gaylord, 2018). Reduction of temperature and desiccation stress enables residents of mussel beds to tolerate low-tide temperature variability under current climate conditions, but also hints at the potential for a dramatic tipping point to become problematic in the future. For example, cascading biodiversity loss could occur if previously noted declines in mussel abundance or density (Smith et al., 2006; Sorte et al., 2017) dip below thresholds necessary for them to support their own recruitment or the success of resident taxa (Jurgens and Gaylord, 2018; see also Sunday et al., 2017).

**Figure 6:**
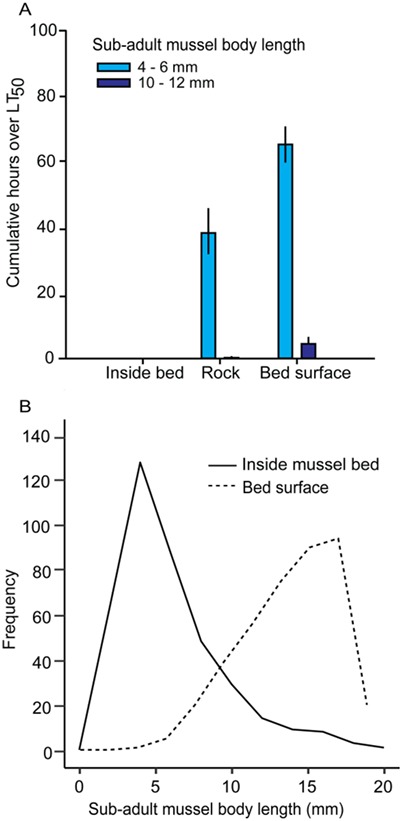
Biophysical interactions can alleviate or exacerbate climate-related stresses for the many taxa that occupy biogenic habitats, but climate risk depends on physiology (e.g. size-specific thermal tolerance) and microhabitat use. (A) Cumulative duration of temperatures over lethal tolerances (here, LT50; the temperature at which 50% of animals die) for two size classes of sub-adult mussels (*Mytilus californianus*) in biogenic (interior and surface locations of mussel beds) and abiotic (rock clearing) habitats. Conditions commonly exceed lethal thermal tolerances for small sub-adults, and occasionally for larger, more heat-tolerant individuals. Data are from 15 months (1 June 2012 to 1 September 2013) of 30-min temperature measurements on horizontal surfaces (loggers: Maxim® DS-1921-G iButtons; *n* = 4 per microhabitat; calibrated, waterproofed in parafilm and coated with marine epoxy). (B) Frequency distribution of sub-adult mussels by size (2-mm bins) as found in the field in within-bed and surface-bed microhabitats at a site in northern California, USA over 1 year (*N* = first 100 individuals sampled per location once per season; seasons pooled). Since larger sub-adults tend to live at the bed surface (B), as climate change exacerbates current conditions, these size classes are more likely to experience major mortality events (despite relatively higher thermal tolerance) than smaller individuals living inside the well-buffered bed. Further experimental details and full dataset available in Jurgens and Gaylord (2016).

Although facilitative effects often dominate the ecological role of mussels, physical effects of high-biomass species aggregations can also vary markedly in space and time. In certain habitat locations, otherwise strongly facilitative species can exacerbate climate-related heat and desiccation stresses. For example, evaluations of the thermal mechanics of the system indicate that aerial temperatures at low tide on the surface of mussel beds can exceed those on adjacent bedrock, likely due to the dark colour and discrete size of the shells that encourages strong solar absorption while also limiting the extent to which heat conducts away into deeper portions of the rock (Jurgens and Gaylord, 2016). Were small size classes of juvenile and sub-adult mussels to position themselves on the bed surface, they would routinely experience heat and desiccation stresses in excess of their thermal tolerances (Fig. 6; Jurgens and Gaylord, 2016). However, the smallest size classes of mussels tend to inhabit the interiors of mussel beds, where conditions are more amenable to survival (Fig. 6). Quantitative understanding of physical feedbacks between climate-driven stressors and within-habitat conditions relevant to associated organisms are therefore critical for predicting population impacts of climate change that stem from physiological stresses (Fig. 6; Jurgens and Gaylord, 2016, 2018).

It is additionally clear that a perspective focused exclusively on low-tide conditions would overlook other factors that can be important for shoreline creatures. In the case of organisms that reside in the interstices of mussel beds, conditions at high tide are strikingly different from those during periods of aerial emergence. This point raises the question of how decreased exchange of mass and momentum might influence chemical parameters of seawater within the interiors of submerged mussel beds. Two types of chemical perturbation have special relevance. First, organisms like mussels and other heterotrophic animals that reside within mussel beds consume dissolved oxygen, making it less available for themselves and other species. Second, the release of CO_2_ associated with respiration and calcification processes (or CO_2_ uptake during photosynthesis by autotrophs; Hurd, 2000; Cornwall et al., 2015) can alter the carbonate system of seawater, exacerbating or potentially offsetting (e.g. Koweek et al., 2018) the consequences of human-produced CO_2_ absorbing into the oceans.

**Figure 7:**
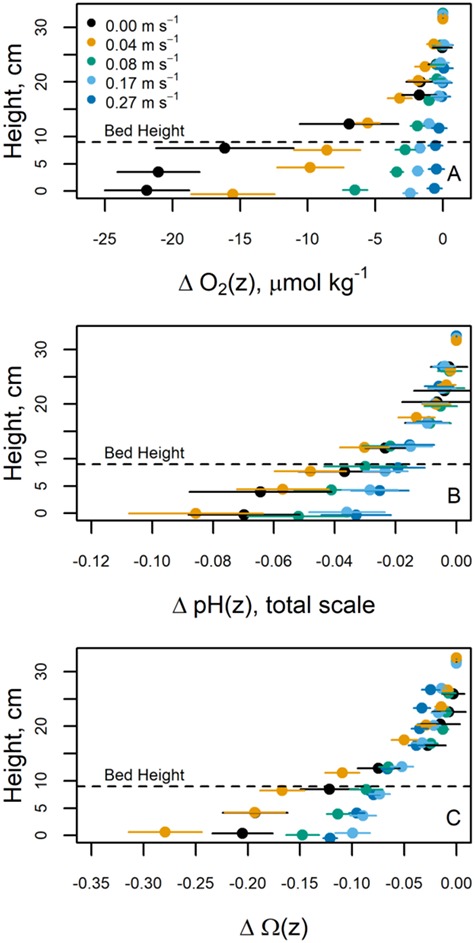
Seawater chemistry can be altered within a mussel bed. Vertical difference profiles for (A) dissolved oxygen, (B) pH (total scale) and (C) calcite saturation state, Ω, across a range of water velocities external to the bed (various colors). Delta symbol indicates differences between chemical parameters at height, *z* (measured upward from the base of the mussel bed), and their corresponding values at the top of each profile. All of these chemical parameters are depressed inside the bed compared to the bulk seawater. Dashed lines indicate the height of the experimental mussel bed. Error bars depict the standard error across multiple profiles within each flow bin. Data have been jittered in the *y*-axis for visibility. Figure modified from Ninokawa et al. (2019).

Thus, although attenuated heat and mass exchange to and from the interior of mussel beds may ameliorate thermal and desiccation stresses for resident organisms at low tide, and although analogous reductions in momentum flux at high tide may provide shelter from large hydrodynamic forces that characterize rocky intertidal habitats (e.g. Denny, 1995; Gaylord, 1999, 2000; Gaylord et al., 2008; Jensen and Denny, 2016), tradeoffs can arise. In particular, elevated chemical stresses can manifest within the interstices of dense aggregations of marine organisms during immersion. For example, we measured seawater conditions in the gaps between mussels within an intact bed transplanted into a flow tank from the field, and did so across a range of flow conditions external to the assemblage. At seawater velocities near zero, the respiration of mussels comprising a bed can decrease oxygen concentrations by approximately 10% (25 μmol kg^−1^) relative to control seawater (Fig. 7; Ninokawa et al., 2019). Likewise, respiration and calcification can lower seawater pH by 0.1 unit compared to surrounding waters. This latter modification corresponds to a decline in the saturation state of calcium carbonate—a factor influencing the ability of many marine calcifiers to produce shells and skeletons (Ries, 2011)—of over 0.25 units (Fig. 7). Note that these chemical changes are applicable to mussel beds in habitats continually refreshed with new seawater, and could be much greater for mussel aggregations within tidepools or other restricted water bodies. The changes in pH and saturation state are also comparable in magnitude to those observed for the open ocean since the preindustrial period. Therefore, seawater conditions within the interstices of immersed mussel beds may cross thresholds for calcification and other physiological processes decades sooner than expected based on projections for the ocean more broadly. The chemical alterations to seawater within the close confines of mussel beds arise most strongly when fluid-dynamic mixing processes are slow, highlighting the importance of ambient flow conditions and interactions between hydrodynamics and the physical structure of the bed. Higher seawater velocities and associated shear and vertical mixing (see, e.g. Gaylord et al., 2004, 2012) will increase rates of water exchange into and out of a mussel bed and will tend to homogenize the interior and exterior seawater chemistry. Likewise, gradients between the interior and exterior of the bed will depend on factors such as the size and packing density of mussels, which also influence how readily mixing occurs. An exploration of such details awaits further attention.

## Summary

The global environment is changing in response to human activities, as evidenced by trends of warming and acidification in the world’s oceans. These shifting conditions have consequences for biomechanical function and behaviour of organisms, together with cascading implications for how individuals and species interact with one another in communities. Although just a first step towards adequate understanding, recent studies of marine taxa on temperate rocky shores reveal the capacity for—and to some extent the scope of—future changes that can be expected due to ocean warming and acidification within these vital ecosystems.
